# Rational design of enzyme activity and enantioselectivity

**DOI:** 10.3389/fbioe.2023.1129149

**Published:** 2023-01-24

**Authors:** Zhongdi Song, Qunfeng Zhang, Wenhui Wu, Zhongji Pu, Haoran Yu

**Affiliations:** ^1^ Key Laboratory of Pollution Exposure and Health Intervention of Zhejiang Province, Interdisciplinary Research Academy, Zhejiang Shuren University, Hangzhou, China; ^2^ Institute of Bioengineering, College of Chemical and Biological Engineering, Zhejiang University, Hangzhou, Zhejiang, China; ^3^ ZJU-Hangzhou Global Scientific and Technological Innovation Centre, Hangzhou, Zhejiang, China

**Keywords:** rational design, enzyme engineering, biocatalysis, enzyme activity, enzyme enantioselectivity

## Abstract

The strategy of rational design to engineer enzymes is to predict the potential mutants based on the understanding of the relationships between protein structure and function, and subsequently introduce the mutations using the site-directed mutagenesis. Rational design methods are universal, relatively fast and have the potential to be developed into algorithms that can quantitatively predict the performance of the designed sequences. Compared to the protein stability, it was more challenging to design an enzyme with improved activity or selectivity, due to the complexity of enzyme molecular structure and inadequate understanding of the relationships between enzyme structures and functions. However, with the development of computational force, advanced algorithm and a deeper understanding of enzyme catalytic mechanisms, rational design could significantly simplify the process of engineering enzyme functions and the number of studies applying rational design strategy has been increasing. Here, we reviewed the recent advances of applying the rational design strategy to engineer enzyme functions including activity and enantioselectivity. Five strategies including multiple sequence alignment, strategy based on steric hindrance, strategy based on remodeling interaction network, strategy based on dynamics modification and computational protein design are discussed and the successful cases using these strategies are introduced.

## 1 Introduction

Almost all reactions in living organisms are catalyzed by protein molecules called enzymes. These enzymes have the potential to be exploited for the synthesis or degradation of chemicals, and in this context, they are often also called biocatalysts. Compared to traditional chemical catalysts, biocatalysts have numerous benefits. Enzymes are mainly produced from microorganisms cultured using renewable resources and are biodegradable and essentially non-hazardous and non-toxic (Sheldon and Woodley, 2018). Additionally, enzyme reactions are generally conducted under relatively mild conditions of pH, temperature and pressure without the need for functional-group activation, protection, and deprotection steps, which are commonly used in conventional organic syntheses. Moreover, the intrinsic chirality of enzymes makes them a powerful tool for the development of stereoselective transformations that are more step-economical and generate less waste compared to organic synthesis (Albarrán-Velo et al., 2017). More importantly, enzymes could be tuned to perform non-natural reactions with high activity and selectivity (Hammer et al., 2017). Due to the above advantages, enzymes have become important tools in various fields including pharmaceuticals, chemical industry, food processing, agriculture, energy and so forth. However, when enzymes are applied in industry to produce high value-added chemicals, the substrates used are often not their natural substrates, resulting in low enzyme activity and enantioselectivity. In addition, the reaction environment of enzymes in industrial applications is different from the physiological environment in which enzymes function in nature. It is often accompanied by high temperature, high pressure, extreme pH, and the presence of organic solvents and various reaction inhibitors (Luetz et al., 2008). These require modifications of enzymes to have improved stability, activity and enantioselectivity for industrial applications.

Directed evolution and rational design are two common strategies used for enzyme molecular modification (Lutz, 2010; Cobb et al., 2013). The strategy of directed evolution was developed in the early 1990s (Bloom and Arnold, 2009). Traditional directed evolution consists of iterative cycles of library construction using random mutagenesis techniques including error-prone PCR, DNA shuffling and site-specific saturation mutagenesis, and the subsequent high-throughput screening for specific functions (Arnold, 1993). Over 30 years of development, directed evolution strategy has been successfully applied for the modifications of enzyme properties such as activity, stability, substrate specificity and stereoselectivity. Due to the contribution to the development of directed evolution, Frances H Arnold was awarded the 2018 Nobel Prize in chemistry (Fasan et al., 2019). Although the directed evolution strategy is powerful, the process of directed evolution is time and labor consuming and requires a high-throughput screening methodology. Additionally, not all enzymes are amenable to developing a high-throughput screening method, nor are all screening methods easy to implement at the required scale.

The rational design strategy is based on the understanding of relationships between enzyme structure and function to predict the potential mutants with desired properties, and to introduce mutations by site-directed mutagenesis. The DNA site-directed mutagenesis (SDM) technique developed by Michael Smith in 1978 makes it possible to investigate the effect of a specific amino acid on protein structure and function, which is of great significance for studying the relationships between protein structure and function (Hutchison et al., 1978; Dalbadie-McFarland et al., 1982; Qu et al., 2018). In recognition of his research, Michael Smith was awarded one-half of the 1993 Nobel Prize in Chemistry. The development of SDM also laid a foundation for the rational design of proteins. For example, based on the mechanism that reducing conformational entropy improves protein stability, Matthews et al. designed Xaa->Pro and Gly->Xaa mutations which successfully improved the stability of T4 lysozyme and maintained the activity as the wild type enzyme (Matthews et al., 1987). Rational design methods are universal, fast and have the potential to be developed into algorithms which can quantitatively predict the performance of the designed sequences (Steiner and Schwab, 2012). However, the limited understanding about enzyme catalytic mechanism and relationship between protein structure and function will affect the accuracy and success rate of rational design strategy.

In recent years, with a considerable increase in the number of protein structures, the improvement of computational power, the development of advanced algorithms, and a deeper understanding of enzyme catalytic mechanisms, there has been an increasing number of studies applying the rational design strategy to engineer enzymes. Compared to the enzyme activity and enantioselectivity, rational design strategies for improving enzyme stability were more readily developed, which include rigidifying flexibility sites (Yu and Huang, 2014), addition of disulfide bonds (Dombkowski et al., 2014; Liu et al., 2016), surface salt bridge engineering (Cui H. et al., 2021), back to consensus mutations (Sternke et al., 2019), calculation of ΔΔG values with Rosetta or FoldX (Yu et al., 2017; Buss et al., 2018) and so forth. And these strategies have been reviewed previously (Pongsupasa et al., 2022). Here, we gave an overview of the latest progress of rational design strategies used to improve the enzyme functions including activity and enantioselectivity. Although it is still challenging to design enzymes with improved activity and enantioselectivity, successful cases have been indeed reported (Table 1). Five strategies including multiple sequence alignment, strategy based on steric hindrance, strategy based on remodeling interaction network, strategy based on dynamics modification and computational protein design are discussed and the successful cases using these strategies are introduced (Table 1). For the strategies based on modifying dynamics and remodeling interaction network, only a few of relative studies relying on solely site-directed mutagenesis have been reported, and studies applying site-saturation mutagenesis were hence also included here to make the approaches better explained and understood.

**TABLE 1:** The rational design strategies to engineer activity and enantioselectivity of enzymes.

Rational design strategy	Properties	Successful cases introduced in this review
Strategy based on multiple sequence alignment	Activity	Bacillus-like esterase Bassegoda et al. (2010), Glutamate dehydrogenase Yin et al. (2018), Amidase Yao et al. (2022), Fructosyltransferase Xia et al. (2022), Adenosylmethionine synthase Wang et al. (2019), Epoxide hydrolase Choi et al. (2012), Aldoxime dehydratase Oike et al. (2021)
	Enantioselectivity	Styrene monooxygenases Liu et al. (2022), Lipase Yi and Park (2021), Epoxide hydrolase Li C. et al. (2020), Li Y. et al. (2020), Carboxylesterases Godinho et al. (2012)
Strategy based on steric hindrance	Activity	Glutamate dehydrogenase Yin et al. (2019), Wang Z. et al. (2022), Dehydrogenase/reductase Li et al. (2016), Su et al. (2020), Alcohol dehydrogenase Wu et al. (2020); Jiang et al. (2022), Epoxide hydrolase Kong et al. (2014), Aldehyde-deformylating oxygenase Bao et al. (2016), Alkane hydroxylase van Beilen et al. (2005), Leucine dehydrogenase Wu et al. (2022), Transaminase Li F. et al. (2022)
	Enantioselectivity	Phosphotriesterase Chen-Goodspeed et al. (2001), Lipase Chen et al. (2014a), Chen et al. (2014b), Yeast old yellow enzyme Wang et al. (2021), Amidase Ao et al. (2021)
Strategy based on remodeling interaction network	Activity	β-amino acid dehydrogenase Liu N. et al. (2021), L-rhamnose isomerase Tseng et al. (2022), Esterase Staudt et al. (2021), Glutamate dehydrogenase Wang Z. et al. (2022)
	Enantioselectivity	P411 enzyme Calvó-Tusell et al. (2022), Lipase CALB Li et al. (2021), Esterase BioH Gu et al. (2015)
Strategy based on dynamics modification	Activity	Kynureninase Karamitros et al. (2022), Alcohol dehydrogenase Liu et al. (2019), Ye et al. (2022), Cytochrome P450 Li Z. et al. (2022), Cumene dioxygenase Heinemann et al. (2021)
	Enantioselectivity	Alcohol dehydrogenase Qu et al. (2021), Lipase CALB Yang et al. (2017)
Computational protein design	Activity	Thioesterase Grisewood et al. (2017), Artificial metalloenzyme Heinisch et al. (2015), Aspartase Li et al. (2018), Cui Y. et al. (2021), Amine transaminase Voss et al. (2018)
	Enantioselectivity	Limonene epoxidase Wijma et al. (2015), Cytochrome P450 Ashworth et al. (2022), Threonine aldolase Zheng et al. (2020)

## 2 Rational design strategy based on sequence alignment

Enzymes with high sequence identity and structural similarity tend to have functional similarity. Based on this, multiple sequence alignment (MSA) has been widely used in engineering enzymes to have improved properties. The straightforward approach is to make mutations in the target enzyme to the corresponding conserved sequence of homologs with desired properties. Furthermore, the CbD (conserved but different) sites, conserved in the pool of homologous sequences but different in the target protein sequence, have been commonly identified to achieve evolutional information from MSA (Yu et al., 2022). Based on the assumption that amino acids appearing with the highest frequency at a particular site among protein sequence homologues tend to contribute more to the protein stability compared to other amino acids, the ‘back to consensus mutations’ approach has been widely applied to engineer stability of enzymes (Steipe et al., 1994; Sternke et al., 2019). Similarly, this method could also be used to improve enzyme functions including activity and enantioselectivity. Different from engineering stability that focuses on mutating regions distant from active sites, engineering activity or enantioselectivity mainly targets on active site region that controls the catalytic properties of enzymes.

### 2.1 Activity

**FIGURE 1:**
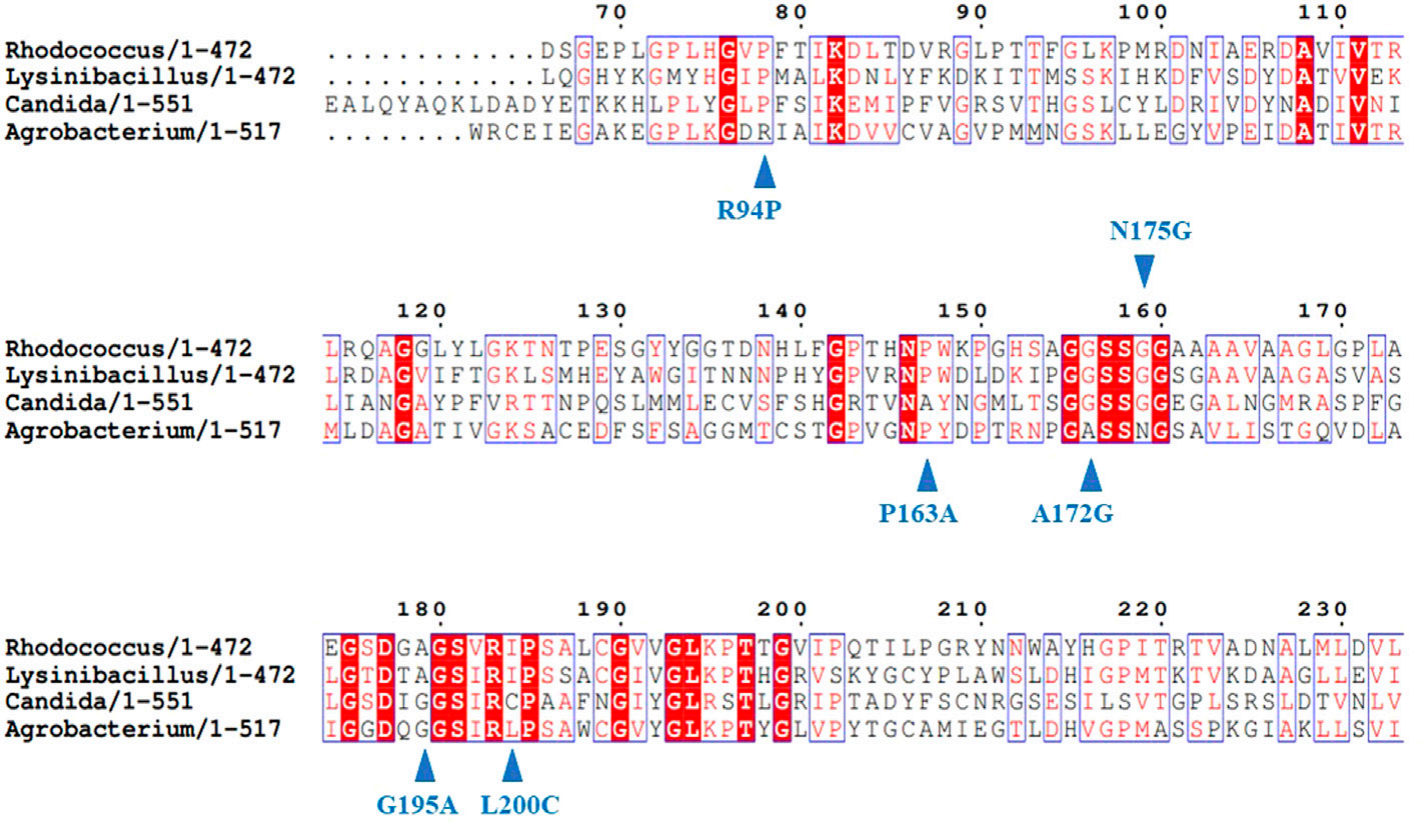
Design of mutations with improved activity using multiple sequence alignment for AmdA. Reprinted with permission from (Yao et al., 2022). Copyright 2022 ACS Publications Division.

Bacillus-like esterase (EstA) has a very low activity in catalyzing the conversion of tertiary alcohol esters whereas its homologous enzymes convert tertiary alcohol esters with high activity. Multiple sequence alignment of 1,343 sequences showed that these enzymes contained a highly conserved GGG motif in the oxyanion hole while EstA contained a serine rather than the conserved glycine at the third position (GGS). After mutating EstA from serine to glycine, the obtained mutant EstA-GGG showed a conversion rate towards tertiary alcohol esters improved by 26 times compared to the wild type (Bassegoda et al., 2010). The similar strategy was also applied to increase the activity of a glutamate dehydrogenase (GluDH) (Yin et al., 2018). Characterization of eight glutamate dehydrogenases obtained through genome mining showed that *Pp*GluDH derived from *Pseudomonas putida* achieved a good soluble expression in *E. coli* and catalyzed the reductive amination of 2-oxo-4-(hydroxymethylphosphinoyl) butyric acid (PPO) to produce L-glufosinate-ammonium with moderate activity. Another glutamate dehydrogenase from *Bordetella prtrii* (*Bp*GluDH) showed a higher activity than *Pp*GluDH, but it failed to achieve complete soluble expression in *E. coli*. After aligning the two sequences, six amino acids that were inconsistent in the two enzyme sequences near the substrate binding pocket were identified. Mutation of the six-amino acid sequence in *Pp*GluDH to the sequence of *Bp*GluDH generated a mutant I170M, which had an enhanced activity by 2.1-fold and maintained a high soluble expression in *E. coli* (Yin et al., 2018). Recently, an amidase from *Agrobacterium tumefaciens* d3 (AmdA) was engineered to have improved activity of degrading carcinogenic ethyl carbamate (EC) in alcoholic beverages with a similar strategy (Yao et al., 2022). The amino acid sequence of AmdA was aligned with three available urethanases showing EC degradation activity from *R. equi* Strain TB-60, *L. fusiformis* SC02, and *C. parapsilosis*. The catalytic triad of AmdA, Lys98-Ser173-Ser197 was conserved in all of the sequences aligned. The CbD sites were identified from regions adjacent to the catalytic triad and the conserved sites in the MSA. Finally, six mutations including R94P, P163A, A172G, N175G, G195A, and L200C were designed based on sequence alignment (Figure 1) and the best mutant G195A improved the activity by 4.9-fold. Moreover, sequence alignment of various fructosyltransferases (FruSGs) indicated that residues for substrate recognition were conserved in all aligned sequences and the CbD residues including Gln38, Ile38, and Cys43 around the active site pocket were identified for mutation. The three residues in FruSG from *Aspergillus niger* were substituted by Tyr and Trp, Met, Asn and Ser, respectively, generating five mutants of Q38Y, Q38W, I39M, C43N, and C43S. Among them, the mutant C43N had a significantly enhanced activity, being 6.9-fold higher compared to the wide type (Xia et al., 2022). In addition to the successful cases described above, this MSA-based rational design strategy has also been applied to engineer the activity of *S*-adenosylmethionine synthase (MAT) from *E. coli* with the variant L186V having reduced product inhibition and 1.5-fold increase in catalytic activity (Wang et al., 2019), epoxide hydrolase (mEH) from *Mugil cephalus* with the hydrolytic activity of the double-point variant E378D/Q170K enhanced by 4.6-fold (Choi et al., 2012), aldoxime dehydratase from *Bacillus* sp. OxB-1 (Oike et al., 2021) and other enzyme molecules.

### 2.2 Enantioselectivity

Sequence alignment could be used to identify the critical residues controlling the enantioselectivity of enzymes. Sequence analysis revealed that (*R*)-selective styrene monooxygenases (SMOs) are clustered in a branch, which is different from the (*S*)-selective SMOs (Liu et al., 2022). Sequence alignment of 9 (*R*)-SMOs and 12 (*S*)-SMOs was applied, identifying13 amino acids that are 100% conserved in (*R*)-SMOs, but different in (*S*)-SMOs. With a highly active (*R*)-SMO from *Streptomyces exfoliates* as the template, the 13 amino acids were then replaced by the residues with the highest frequency at the corresponding sites in (*S*)-SMOs. Four of the mutants displayed higher enantioselectivity than the wild type and the best one-W86I showed 98% *ee,* while variant A219V displayed inverted stereoselectivity yielding (*S*)-styrene oxide with 43% *ee*. Saturation mutagenesis at these two sites led to mutants with further improvement in the enantioselectivity and when these mutations were transferred to other (*R*)-SMOs, the similar effect was achieved (Liu et al., 2022). Sequence alignment with homologues having high enantioselectivity could also be used to guide the enantioselectivity engineering of a target enzyme. Lipase B from *Candida antarctica* (CAL-B) exhibited low enantioselectivity towards *sec*-alcohols with substituents smaller than a propyl group while its homologous enzyme PBL, lipase from *Pseudozyma brasiliensis*, displayed high enantioselectivity towards (±)-but-3-yn-2-ol. Following alignment of the two sequences sharing 72% identity, the local different residues 42, 43, 45, and 47 in the medium binding pocket were identified and the sequence of residues 42–47 of CAL-B was then replaced with that of PBL. The generated mutant CAL-B-42–47 exhibited improved enantioselectivity by 40–50 times towards (±)-but-3-yn-2-ol and by 3–4 times towards (±)-butan-2-ol compared to the wild type (Yi and Park, 2021). Significant improvement in the enantioselectivity of an epoxide hydrolase from *Sphingomonas* sp. HXN-200 (*Sp*EH) was also observed after multiple site-directed modification for hydrolysis of *rac*-phenyl glycidyl ether (*rac*-PGE). Multiple sequence alignment of 64 EHs was conducted, leading to the identification of six residues V195, V196, F218, N226, Q312, and M332 near the active site as targets. These non-conservative residues were mutated to hydrophobic amino acids by site-directed mutagenesis. The resulting optimal *Sp*EH triple mutant V196A/N226A/M332A exhibited 2.8-fold higher enantioselectivity towards *rac*-PGE, which had a greater affinity towards *S*-PGE than *R*-PGE compared to the wild type (Li Y. et al., 2020). Additionally, successful application of the sequence alignment based rational design strategy in improving enantioselectivity has also been reported for other enzymes such as a *Phaseolus vulgaris* epoxide hydrolase (*Pv*EH2) with the enantioselectivity of variant *Pv*2*St* towards *rac*-1,2-epoxyhexane significantly increased by 11.5-fold (Li C. et al., 2020), a carboxylesterase from *Bacillus subtilis* (CesA) with the mutant F166V/F182C showing an increased enantioselectivity by 13-fold towards 1,2-O-isopropylideneglycerol (IPG) butyrate (Godinho et al., 2012), and so forth.

## 3 Rational design strategy based on steric hindrance

Spatial hindrance refers to the mutually exclusive effect when the distance between two or more atoms and chemical groups is smaller than the van der Waals radius. When using an enzyme to catalyze a non-natural substrate, the size and shape of the substrate may not match the substrate access tunnel or binding pocket of the enzyme, where the steric hindrance will occur. The steric hindrance would result in unstable interactions between the substrate and the enzyme, leading to lower enzyme activity or enantioselectivity. Hence, if the substrate binding pocket or molecular tunnel of enzyme is modified to better bind substrate or release product, the enzyme functions could be improved.

### 3.1 Activity

**FIGURE 2:**
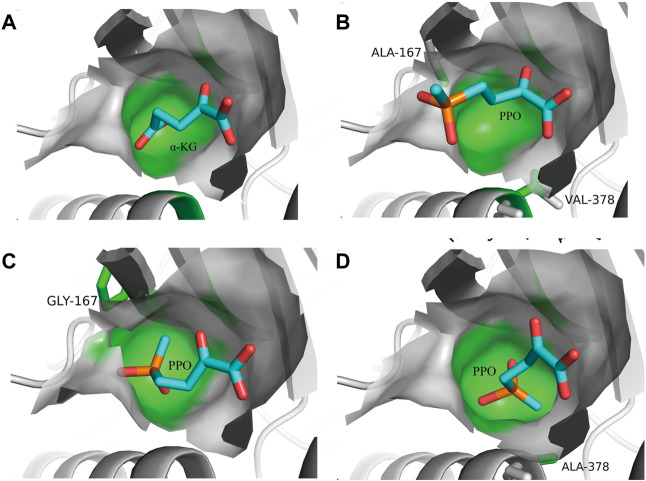
Rational design of glutamate dehydrogenase based on steric hindrance. **(A)**, *Pp*GluDH docked with native substrate α-KG. **(B)**, *Pp*GluDH docked with non-natural substrate PPO. **(C)**, *Pp*GluDH variant A167G docked with non-natural substrate PPO. **(D)**, *Pp*GluDH variant V378A docked with non-natural substrate PPO. Reprinted with permission from (Yin et al., 2019). Copyright 2019 Wiley-VCH Verlag GmbH & Co. KGaA, Weinheim.

**FIGURE 3:**
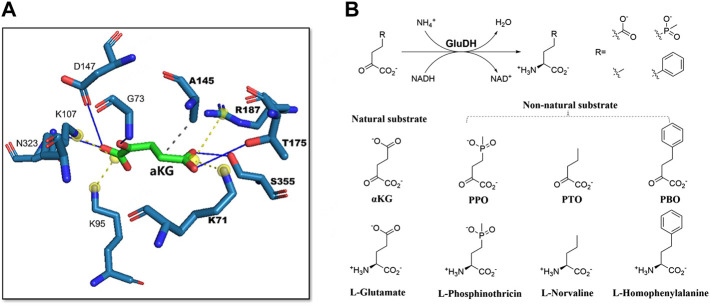
Engineering of glutamate dehydrogenase for accepting non-natural substrates. **(A)**, Salt bridges formed between γ-carboxyl group of the natural substrate αKG and two residues K71, R187. **(B)**, The natural and non-natural substrates of GluDH and the corresponding products. Reprinted with permission from (Wang Z. et al., 2022). Copyright 2022, American Chemical Society.

Several studies have reported the successful application of steric hindrance based rational design strategy in enzyme activity enhancement towards bulky substrates (Li F. et al., 2022; Wang Z. et al., 2022; Wu et al., 2022). α-ketoglutarate (α-KG) is the natural substrate of glutamate dehydrogenase (GluDH). When GluDH was used to catalyze a bulky non-natural substrate 2-oxo-4-[(hydroxy) (methyl) phosphinyl]-butyric acid (PPO) in a reductive amination reaction, the enzyme activity decreased significantly. The main difference between PPO and α-KG is the methyl phosphinyl group at the γ position in PPO compared to the γ-carboxyl group in α-KG (Figure 2A, B). The results of molecular docking showed that due to steric hindrance, the large methyl phosphinyl group of PPO could not enter the substrate binding pocket of *Pp*GluDH, which prevented PPO binding and resulted in a decreased catalytic activity. Therefore, it is possible to optimize the PPO binding by expanding the substrate binding pocket of *Pp*GluDH, thereby increasing the activity of *Pp*GluDH towards PPO. Five amino acid residues with larger side chains including Lys93, Thr196, Arg208, Val378, and Ser381 in the binding pocket were mutated to alanine with a smaller side chain, and the residue of Ala167 was replaced with a smaller glycine. It was found that two mutations of A167G and V378A (Figure 2C, D) significantly increased the catalytic activity of *Pp*GluDH towards PPO with the measured specific activity of purified enzyme reaching 38.13 U/mg and 35.96 U/mg respectively, being 123-fold and 116-fold higher than that of the wild type (Yin et al., 2019). Recently, a GluDH from *Clostridium difficile* 630 (*Cd*GluDH) was engineered with the similar strategy to reduce the steric hindrance in substrate binding pocket, leading to significantly improved enzyme activity of variants A145G, P144A, and V143A towards non-native bulky substrates of 2-oxo-4phenylbutyric acid (PBO) and PPO for synthesizing L-homophenylalanine and L-phosphinothricin, respectively (Figure 3) (Wang Z. et al., 2022). The rational design strategy based on steric hindrance has also been successfully applied in the modification of enzymes such as dehydrogenase/reductase EbSDR8 (Li et al., 2016; Su et al., 2020) and alcohol dehydrogenase (Wu et al., 2020; Jiang et al., 2022). Active pocket iterative mutagenesis was performed by replacing the rationally selected five residues around the catalytic triad of an alcohol dehydrogenase from *Lactobacillus kefiri* (*Lk*ADH) with smaller glycine, alanine, cysteine, serine, proline and valine. A final mutant Y190P/I144V/L199V/E145C/M206F was obtained, exhibiting increased activity and excellent stereoselectivity towards diaryl ketones by eliminating steric hindrance in the binding pocket and constructing more non-covalent interactions (Wu et al., 2020). In another study, an ADH from *Thermoanaerobacter brockii* (*Tb*SADH) was engineered by targeting a remote residue H42 that functions in proton transfer. The resulting mutant H42T showed a 9-fold enhanced activity towards a bulky prochiral ketone due to the expanded substrate-binding pocket (Jiang et al., 2022).

Besides targeting on the substrate binding pocket, engineering to eliminate steric hindrance in the molecular tunnel can also increase enzyme activity (Gautieri et al., 2022). Natural epoxide hydrolase has very low activity when catalyzing bulky epoxide substrates. With an epoxide hydrolase from *Bacillus megaterium* (*Bm*EH) as a model, X-ray crystal structure determination, mass spectrometry and molecular dynamics simulations were applied to illustrate the enzyme catalytic mechanism and identify the substrate access and product release channels. Subsequently, large-volume leucine, methionine, and phenylalanine in the molecular channel were mutated to small-volume alanine to extend the potential product-release tunnel. Two mutants F128A and M145A were obtained, exhibiting expanded product-release site and increased activities towards bulky α-naphthyl glycidyl ether by 42 and 25 times, respectively compared to the wild type (Kong et al., 2014). In addition, the substrate specificity of enzymes can be adjusted by modifying the molecular channel as well. Large-volume amino acids were introduced into the substrate channel of an aldehyde-deformylating oxygenase, which introduced steric hindrance and narrowed the substrate channel, thereby improving the enzyme activity towards short-chain aldehyde substrates (Bao et al., 2016). Similarly, an alkane hydroxylase from *Mycobacterium tuberculosis* was introduced with large-volume amino acids into the substrate tunnel, making it oxidize only shorter alkanes (van Beilen et al., 2005).

### 3.2 Enantioselectivity

The rational design strategy based on steric hindrance has also been used in improving enantioselectivity of enzymes. The binding pocket of enzyme can be modified according to the size and chirality of the substrate to reduce the energy loss during substrate binding, thereby increasing the reaction rate towards the target substrate and the enantioselectivity of enzyme. This strategy has been successfully used to modify the enantioselectivity of a phosphotriesterase (PTE) towards organophosphotriesters (Chen-Goodspeed et al., 2001). The wild-type PTE accepted the *Sp-*enantiomers at a rate 10 times higher than the rate at which it accepted the *Rp*-enantiomers. In the study, it was found that reduction in the size of substrate-binding pocket by G60A mutation increased the enantioselectivity of the enzyme for the preferred *Sp-*enantiomers. On the other hand, expanding the size of the substrate-binding pocket by I106G, F132G, and S308G mutations significantly enhanced the enzyme stereoselectivity for the *Rp*-enantiomers by up to 2700 times (Chen-Goodspeed et al., 2001). Additionally, improvement in the enzyme enantioselectivity could also be achieved by using steric hindrance to favor the target substrate binding and prevent non-target substrates from binding to the active site. A lipase from *Pseudomonas alcaligenes* (*Pa*L) that hydrolyzes L-menthyl propionate to produce L-menthol with high stereoselectivity was engineered for diastereoselectivity using the steric hindrance based rational design strategy (Chen et al., 2014a; Chen et al., 2014b). (2*S*,5*R*) L-menthol is an important chemical, widely used in manufacturing oral hygiene products. Menthyl propionate contains three chiral centers and eight isomers, and only hydrolysis of the (2*S*,5*R*) L-menthyl propionate could produce the target product (2*S*,5*R*) L-menthol. Although *Pa*L has an excellent resolution effect on D-, L-menthyl propionate and hardly hydrolyzes D-menthyl propionate, its diastereomeric selectivity is not ideal. Besides recognizing (2*S*,5*R*) L-menthyl propionate, (2*R*,5*S*) L-neomenthyl propionate and (2*R*,5*R*) D-isoneomenthyl propionate, *Pa*L also reacts in small amounts with three other menthyl propionate configurations. Structural analysis of the docked complexes of *Pa*L and the three substrates showed that there were cavities around the stereocenters of substrates, which led to the low diastereoselectivity of *Pa*L. Mutations of residues V180 and A272 which interact with substrates to larger amino acids filled the cavity and limited the orientation of the target substrate. The increased steric exclusion and decreased structural flexibility of the catalytic pocket resulted in less binding of non-target substrates, thereby increasing the diastereoselectivity of *Pa*L. The double point mutation V180L/A272F effectively increased the diastereoselectivity towards (2*S*,5*R*) L-menthyl propionate by 4.7 times (Chen et al., 2014a). The yeast old yellow enzyme from *Saccharomyces cerevisiae* S288C (OYE3) could catalyze the reduction of (*E/Z*)-citral for direct production of (*R*)-citronellal, but exhibited low enantioselectivity. In order to obtain the enantio-pure (*R*)-citronellal, semi-rational design strategy was used to modify the substrate binding and utilization modes of the OYE3 to improve its (*R*)-enantioselectivity. It was found that mutation of the residue W116 to larger amino acids decreased the (*R*)-enantioselectivity while replacing W116 with smaller residues enlarged substrate binding space and favored a flipped binding mode for (*Z*)-citral, thereby promoting the (*R*)-enantioselectivity to >99%. A final double point mutant S296F/W116G was obtained, exhibiting strict (*R*)-enantioselectivity towards (*E*)-citral and (*E/Z*)-citral without any (*Z*)-citral catalytic activity (Wang et al., 2021). The rational design strategy based on steric hindrance can also be used to modify enzyme enantioselectivity in biocatalytic desymmetrization of meso substrates. Recently, rational engineering of an amidase from *Rhodococcus erythropolis* by adjusting steric hindrance successfully reversed and enhanced its enantioselectivity to 99% in desymmetrization of meso heterocyclic dicarboxamides for synthesizing both desired antipodes of products. Structural analysis showed that the steric hindrance in the catalytic cavity limited the binding of *S*-amide group of substrates to the active site, leading to the *R*-enantiopreference of amidase towards the six-membered substrate. By mutating the I450 in the back area of catalytic cavity to a smaller G, the F146 in the entrance tunnel to a smaller A and the A332 in the tail area to a larger F or W, a variant I450G/A332W/F146A was obtained, achieving completely reversed *S*-enantiopreference with high efficiency. Mutation of the G193 close to *R*-amide group to a smaller A significantly increased the *R*-enantioselectivity of amidase towards the five-membered substrates (Ao et al., 2021).

## 4 Rational design strategy based on remodeling interaction network

The active site of enzyme is the core region where the complete catalysis cycle is performed including substrate binding, formation of the transition state, and then product release (Toscano et al., 2007). The interactions such as hydrogen bonds, hydrophobic interactions, salt bridges and so forth formed between substrates and the enzyme active site play a crucial role in anchoring the substrates to a correct position ready for catalytic reaction. Hence, it could be a useful strategy to rationally remodel the interaction network involved between the substrates and enzyme active site to modify the substrate binding affinity and hence enhance the enzyme activity and enantioselectivity.

### 4.1 Activity

Clear understanding of the enzyme catalytic mechanisms and substrate binding is important for accurate design of mutants with improved properties. β-amino acid dehydrogenase (β-AADH) is a promising enzyme used for the asymmetric synthesis of chiral β-amino acids. However, the only reported β-AADH, *L-erythro*-3,5-diaminohexanoate dehydrogenase (3,5-DAHDH), showed strict substrate specificity towards native substrate, which limited the applications. Recently, the crystal structures of 3,5-DAHDH from *Candidatus Cloacamonas acidaminovorans* and its variant were determined and quantum mechanics/molecular mechanics (QM/MM) was carried out to illustrate its catalytic mechanism (Liu N. et al., 2021). Based on the crystal structures and catalytic mechanisms explored, rational design strategy was applied to engineer a previous variant E310G that significantly affected substrate scope to have enhanced activity towards non-native substrates (Zhang et al., 2015). Glu310 in the wild type formed a hydrogen bond with the amide of NADPH, responsible for binding the cofactor. However, the E310G mutation destroyed the binding due to the absence of a hydrogen bond. It was hence hypothesized that the interaction between the amide of NADPH and enzyme could be reconstructed thereby improving the catalytic activity of E310G mutant. Since the C_α_ atom of Gly323 was the nearest, 3.8 Å away from the amide group of NADPH, it was firstly mutated to serine with an expectation to form a hydrogen bond between the short side-chain hydroxyl group of serine and the amide group of NADPH. As a result, the mutant E310G/G323S showed an improved specific activity towards target substrate by 17-fold compared to its parent enzyme E310G (Liu N. et al., 2021). Furthermore, a _L_-rhamnose isomerase from *Thermoanaerobacterium saccharolyticum* (*Ts*RhI) was engineered to have altered substrate specificity from L-rhamnose to D-allose by reconstructing the interactions between substrate and enzyme active site. Since I102 might be involved in the substrate recognition and has a noticeable shorter distance to the substrate compared to other amino acids, it was mutated to ten other polar or charged amino acids in an attempt to obtain stronger interactions between *Ts*RhI mutants and the target substrate D-allose. The results of activity screening against different substrates showed that I102N, I102Q, and I102R variants significantly increased the substrate preference towards D-allose with the catalytic efficiencies being 148%, 277% and 191% respectively, compared to the wild-type. The increased activity of I102Q could be attributed to the introduction of a hydrogen bond, which stabilized the enzyme-allose complex and increased the substrate affinity to the enzyme (Tseng et al., 2022). In addition, a rational design strategy based on remodeling the hydrophobic interactions has also been applied to improve the acyltransferase activity of an esterase from *Pyrobaculum calidifontis* VA1 (PestE). Biocatalytic transesterification is commonly conducted using lipases under anhydrous conditions as hydrolases usually favor hydrolysis over acyl transfer in bulk water. In order to improve the acylation activity of PestE for synthesizing monoterpene esters in an aqueous solution, residues composing the binding of substrate (–)-menthol in the active site were examined. The increase in the hydrophobicity of active center can provide a more favorable surrounding for organic nucleophiles than for water. Three residues including His95, Ile208, and Asn288 were hence selected for rational protein engineering aiming to increase active site hydrophobicity and tunnel size (Staudt et al., 2021). Three variants including H95A, I208A and N288F were constructed. Among them, the mutant I208A was highly enantioselective for (–)-menthyl acetate, and N288F exhibited good acyl transfer activity in an aqueous medium with low hydrolysis of the formed monoterpene esters at the same time. Recently, a glutamate dehydrogenase from *Clostridium difficile* (*Cd*GluDH) was engineered to have improved activity towards non-native substrate 2-oxopentanoic acid (PTO). When the enzyme binds with the nature substrate α-ketoglutarate (α-KG), two salt bridges are formed between γ-carboxyl of α-KG and two residues Lys71 and Arg187 (Figure 3A). However, the side-chain of PTO possesses a non-polar group at the γ position and would not form any electrostatic interactions with the polar residues (Figure 3B). The residues Lys71 and Arg187 were hence mutated to hydrophobic alanine to modify the polarity of the binding pocket. As a result, L187A mutant failed to improve the enzyme activity, whereas the variant K71A improved the enzyme activity by 2.3-fold compared to the wild type (Wang Z. et al., 2022).

### 4.2 Enantioselectivity

**FIGURE 4:**
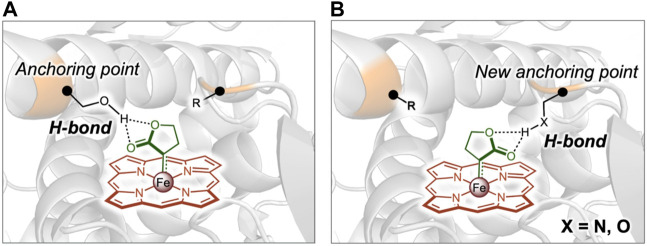
Reversing the enantioselectivity of enzymatic carbene N–H insertion by remodeling the interaction network. **(A)**, Hydrogen bond shown in enzyme variant with high enantioselectivity towards *S*-enantiomer. **(B)**, Newly constructed hydrogen bond to invert the enantioselectivity towards *R*-enantiomer. Reprinted with permission from (Calvó-Tusell et al., 2022).

The enantioselectivity of enzyme could be rationally designed based on modifying the interactions between substrates and enzyme catalytic site. Recently, mechanism-guided rational engineering of a P411 enzyme was conducted to have inverted enantioselectivity by introducing a new hydrogen-bond anchoring point in the enzyme active site (Calvó-Tusell et al., 2022). Previous engineering of P411 enzymes enables insertion of lactone-carbene (LAC) into carbene N-H for the asymmetric synthesis of chiral amines with high efficiency and enantioselectivity (Liu Z. et al., 2021). A variant L7 catalyzed the carbene N-H bond insertion for efficient amine production with up to 99:1 *er* towards the *S*-enantiomer and it was found that the LAC intermediate in the *S*-selective L7 variant was anchored by a hydrogen bond formed between the lactone ester group and the side chain of a critical Ser264, which controlled the stereoselectivity. Therefore, it was hypothesized that the orientation of the LAC could be modulated to invert L7 enantioselectivity by substituting the Ser264 with a non-polar alanine residue to disrupt the hydrogen bond interaction, and meanwhile introducing a new hydrogen bond donor residue serving as a new LCA anchoring point at the opposite of Ser264 in the binding pocket (Figure 4). By analyzing the computational models generated for an unselective variant and a *S*-selective variant, both with the LAC bound, two positions 268 and 328 were identified to be potential anchoring points of the new hydrogen bond. The S264A mutation was then constructed and site-directed saturation mutagenesis was carried out at the 328 and 268 sites of the *S*-selective variant. Following screening, two variants V328Q and V328N were generated, which improved the enantioselectivity towards the *R*-enantiomer to 9:91 and 7:93 *er*, respectively. The molecular dynamics simulations showed that, as expected, the mutant V328N formed a stable hydrogen bond with the ester group of the LAC, which was exactly placed at the opposite side in the catalytic pocket compared to Ser264 in the *S*-selective variant (Calvó-Tusell et al., 2022). Strengthening electronic interactions between substrate and enzyme active site was also used to improve the enantioselectivity of *Candida antarctica* lipase B (CALB) (Li et al., 2021). It was difficult for CALB to catalyze the hydrolytic kinetic resolution (KR) of bulky racemic phenyl(pyridin-4-yl) methyl acetate due to the steric effect of Trp104. The previously reported mutant W104A enlarged the binding pocket and achieved a successful KR with good yield and *S*-enantioselectivity of 91% *ee*. The *S*-selectivity of W104A was probably due to the electronic effect between polar residues in binding pocket and pyridyl of substrate. An electronic effect-guided rational design strategy was thus employed to further enhance the *S*-selectivity of CALB. Three polar amino acids including Cys, Ser and Thr with similar size as alanine were introduced at the 104 position to reconstruct the substrate binding pocket with increased polarity, thereby increasing the interactions between polar side chains of residues at 104 and N atom of pyridine-4-yl in the substrate. As a result, the mutants W104C and W104T improved the *S*-enantioselectivity from 91% to 99% and 98%, respectively, with the similar yield compared to the mutant W104A (Li et al., 2021). Similarly, the substrate binding was rationally modified by simultaneously tuning electronic interactions and steric effects, leading to up to 22-fold enhancement in the enantioselectivity of an esterase BioH towards methyl (*S*)-o-chloromandelate (*S*-CMM) (Gu et al., 2015).

## 5 Rational design strategy based on dynamics modification

The entire enzyme catalytic cycle consists of substrate binding, substrate-enzyme complex formation for catalysis and product release processes, which involves enzyme conformational opening and closing. Dynamic motion of enzymes occurs over a wide range of timescales and large conformational changes during catalysis have been observed for many enzymes, the pivotal role of which in modulating all steps of the catalytic cycle is being increasingly understood. Enzymatic catalysis requires not only the catalytic pocket, but also the dynamic network associated with the catalytic pocket, substrate or product channels, and domains that coordinate changes in conformation. Hence, rational or semi-rational design strategy based on dynamics modification could be used to tune the enzyme functions.

### 5.1 Activity

**FIGURE 5:**
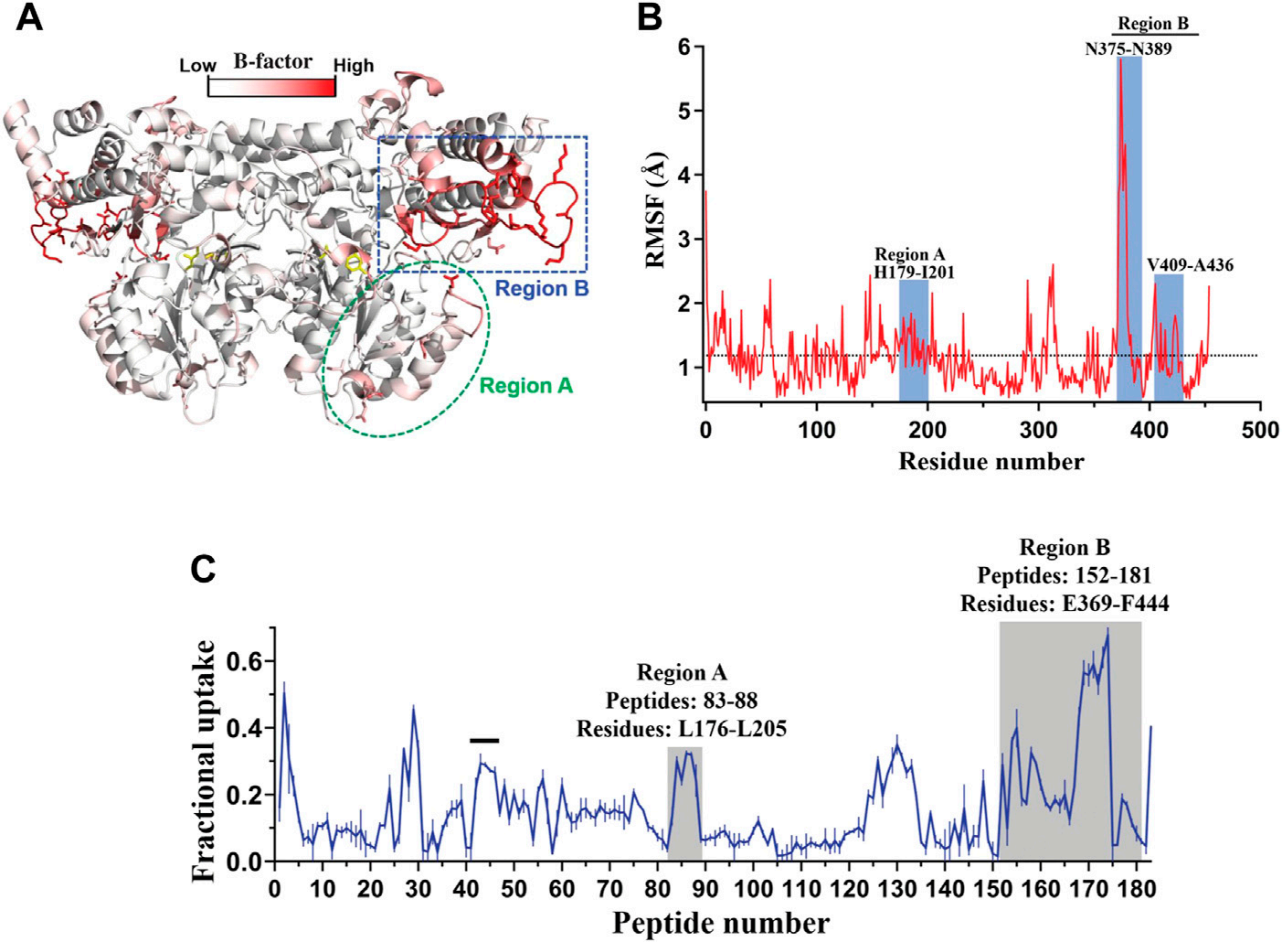
Flexibility analysis of HsKYNase based on B-factor, MD simulations and HDX-MS. **(A)**, The protein structure colored by B-factor values in HsKYNase. **(B)**, Calculated RMSF values of HsKYNase residues obtained and averaged from MD simulations. **(C)**, D_2_O uptake of HsKYNase after 1 min exposure in the absence of ligand. Reprinted with permission from (Karamitros et al., 2022). Copyright 2022 National Academy of Sciences.

The strategy of rigidifying flexible sites guided by phylogenetic analysis (Watanabe et al., 2006), molecular dynamics (MD) simulations, RosettaDesign (Lee et al., 2020), or FoldX (Huang et al., 2017) has been widely used for improving protein thermal stability. Interestingly, it has been observed that the mutagenesis of residues located at flexible regions remote from the active center could also significantly improve the enzyme catalytic efficiency. Recently, such a method was applied to engineer *Homo sapiens* kynureninase (HsKYNase) for increasing its catalytic activity towards a non-preferred substrate, L-kynurenine (KYN) (Karamitros et al., 2022). B-factor analysis, hydrogen-deuterium exchange coupled to mass spectrometry (HDX-MS) and MD simulations were first applied to predict the highly flexible regions distal to the active center (Figures 5A–C–C). Subsequently, saturation mutagenesis libraries were constructed at two to five non-conserved amino acid sites in the two identified flexible regions and screened for improved variants. A combined variant BF-HsKYNase was obtained, exhibiting 18.3-fold higher catalytic efficiency (*k*_cat_/*K*_m_) against the target substrate KYN and 7.5-fold lower *k*_cat_/*K*_m_ towards native substrate OH-KYN compared to the wild type. Further analysis by HDX-MS and MD simulations revealed that the distal mutations in BF-HsKYNase allosterically influenced the flexibility of the cofactor pyridoxal-5′-phosphate (PLP) binding pocket. This probably altered the conformational ensemble and led to sampling states more favorable to the catalyzed reaction, thereby affecting the reaction rate (Karamitros et al., 2022). Additionally, a conformational dynamics-guided loop engineering strategy was employed to improve activity of an alcohol dehydrogenase from *Thermoanaerobacter brockii* (TbSADH) towards a non-native substrate (Liu et al., 2019). Five amino acids at the binding pocket including A85, I86, W110, L294 and C295 involved in modulating activity were explored for structural flexibility and it was found that A85, I86, L294 and C295 were relatively rigid, which might impose restrictions on substrate recognition. I86, L294, and C295 were thus *in silico* replaced by alanine, while A85 was substituted with glycine. MD simulations analysis indicated the clear changes in RMSF values of A85G and I86A compared to the wild type, reflecting that mutations at A85 and I86 were more likely to induce fluctuations in conformational dynamics of the active site loop. The two residues were then subjected to site-directed saturation mutagenesis and several mutants with improved activity towards the target difficult-to-reduce bulky ketone were obtained. Further screening of the mutation library combining A85 and I86 led to the best variant A85G/I86L, successfully reducing the target ketone to (*S*)-alcohol with 99% *ee* at 98% conversion due to the improved loop flexibility (Liu et al., 2019). Recently, a medium-chain alcohol dehydrogenase was engineered for efficient synthesis of (*S*)-N−Boc-3−pyrrolidinol by adjusting the conformational dynamics of loops (Ye et al., 2022). Similarly, highly flexible loops surrounding the tunnel entrance of a cytochrome P450 were engineered to enhance its substrate access. The best variant obtained reduced the flexibility of two critical loops, responsible for improving the stability of the substrate access tunnel, and showed 134-fold improvement in the catalytic activity (Li Z. et al., 2022). A loop engineering strategy was also applied to improve the activity and selectivity of a cumene dioxygenase from *Pseudomonas fluorescens* IP01. With the two highly flexible active-site loops as the mutation targets, screening of mutants constructed based on alanine scanning, sequence alignment, and novel loop insertions along with deletions (InDels) resulted in variants showing up to 16-fold increase in activity. InDels were demonstrated to affect the loop dynamics in terms of flexibility and length, resulting in variants with improved activity or selectivity (Heinemann et al., 2021).

### 5.2 Enantioselectivity

The conformational dynamics of active site also play an important role in manipulating the enantioselectivity of enzymes. Recently, a proline-induced loop engineering strategy was applied in order to trigger fluctuations in dynamics of an active site loop for improved stereoselectivity in TbSADH (Qu et al., 2021). A flexible loop region at 84–92 positions adjacent to the β6 strand is observed close to the substrate binding pocket in TbSADH. Interestingly, the loop contains an interfaced proline at position 84, resulting in dramatical conformational rigidity in the loop. Saturation mutagenesis and site deletion (ΔP84) were then performed at this position to manipulate the loop dynamics. MD simulations revealed that five mutants including P84G, P84S, P84V, P84Y and ΔP84 displayed significant increase in flexibility indicated by improved RMSF values of residue 84 and the whole loop region when compared to the wild type. The following experiments showed that mutants P84S and P84Y gained (*S*)-selectivity with modest conversion whereas the ΔP84 mutant exhibited (*R*)-selectivity. Combinational saturation mutagenesis was then performed at two positions 85 and 86 with P84S as template for (*S*)-selectivity and ΔP84 as the (*R*)-selective template, respectively. As a result, two double variants P84S/I86L and P84S/I86A displayed high (*S*)-selectivity of >99% *ee* at > 99% conversion. And one double mutant ΔP84/A85G showed high (*R*)-selectivity of 97% *ee* at 99% conversion. Further MD simulations indicated a higher loop movement at residues 84 and 85 in the two best variants P84S/I86L and ΔP84/A85G compared to WT, which enlarged substrate binding pocket and increased the plasticity of substrate binding pocket (Qu et al., 2021). MD simulation is a key approach to investigate the flexibility of a protein and could be used for screening variants with flexibility modified. Lipase CALB was subjected to conformational dynamics engineering for improved *R*-enantioselectivity with the utility of MD simulations (Yang et al., 2017). The previously obtained CALB variant, EF5, showed high *R*-enantioselectivity of 98.5% *ee* at 5°C, but a low *R*-ee value of only 8% at 30°C. Conversely, the catalysis yield increased from 13.2% to 80% when temperature increased from 5°C to 30°C. MD simulations revealed an increase in the conformational dynamics of the active pocket and tunnel triggered by high temperature, and modulating the dynamics was hence proposed to increase the *R*-enantioselectivity at 30°C. Thirteen residues in substrate binding pocket and channel were selected for in silico alanine scanning or substituting serine for original alanine, resulting in the identification of two key residues D223 and A281, as indicated by clear changes in RMSFs for D223A and A281S. The two key sites were then subjected to saturation mutagenesis computationally for identifying variants with reduced RMSFs and two mutants D223V and A281S were screened out. The two single variants and their combined mutant D223V/A281S were then constructed for experimental evaluation. It turns out that the *R*-enantioselectivity increased from 8% *ee* for parent EF5 to 93.5% *ee* for A281S, 95.8% *ee* for D223V, and >99% *ee* for D223V/A281S at 30°C, reflecting that decreasing the conformational dynamics of pocket and channel was a useful strategy to improve *R*-enantioselectivity of CALB (Yang et al., 2017).

## 6 Computational protein design

Computer-aided protein design is based on the assumption that the conformation of protein molecules is always at the lowest energy conformational state in the amino acid sequence space observed in nature. It relies on accurate energy functions and reasonable conformational sampling methods to fulfill the protein design. The lower the binding energy between substrate and enzyme molecule is, the better the substrate interacts with the enzyme active site. The reactivity of enzyme could hence be improved by designing mutations to maintain the correct configuration of the substrate at the active site, and reducing the binding energy of the substrate to the active site. This process requires the assistance of a variety of computational tools and methods such as homology modeling, molecular docking, MD simulation, quantum mechanical simulation, and Monte Carlo simulated annealing. Although it remains a challenge to computationally design an enzyme with improved activity or enantioselectivity, the number of successful cases is increasing.

### 6.1 Activity

**FIGURE 6:**
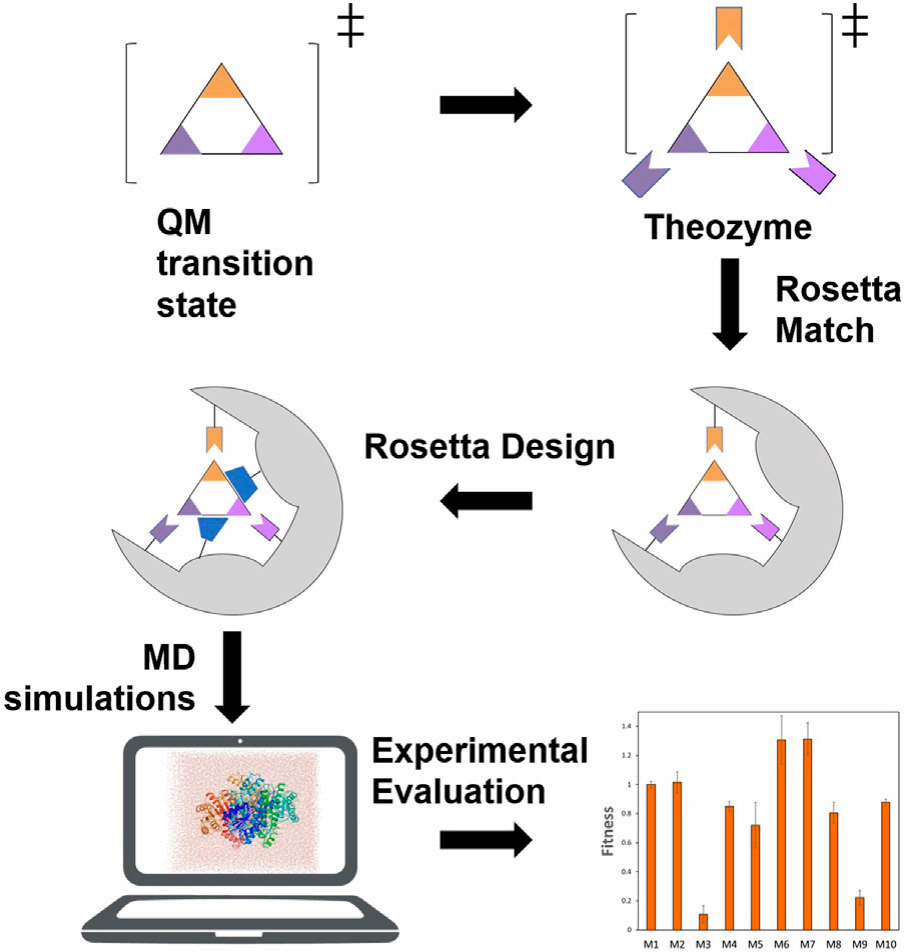
A representation of an enzyme *in silico* design protocol. The full steps including calculation of the theozyme model, insertion of the reaction transition state into a protein scaffold, Rosetta design to optimize the sequence of the enzyme, MD simulations for virtual screening and experimental evaluation.

The binding energy between substrate and enzyme active site could be used as an indicator of fitness to optimize enzyme activity. The substrate binding pocket of thioesterase has been redesigned to improve its catalytic activity against medium and long-chain fatty acids (Grisewood et al., 2017). A reciprocating protein design and optimization algorithm (Iterative Protein Redesign and Optimization, IPRO) (Pantazes et al., 2015) was used to design and optimize the protein in multiple rounds until the binding ability of the substrate was significantly enhanced, and finally 37 mutants were obtained with significant improvements in catalytic efficiency towards C_8_ fatty acid (Grisewood et al., 2017). Rosetta software has been used to modify various functional properties of enzymes, including substrate specificity (Liu et al., 2014), stereoselectivity (Li et al., 2018) and so forth. The Rosetta *de novo* enzyme design process generally includes stages: calculation of the theozyme model, insertion of the reaction transition state into a protein scaffold, Rosetta design to optimize the sequence of the enzyme, MD simulations for virtual screening and experimental evaluation (Figure 6). This process involves using the Monte Carlo algorithm to search for the amino acid sequence and side chain conformations surrounding the ligand, or using Foldit to introduce mutations, and using the Rosetta energy function to calculate the total energy of protein and the binding energy of the enzyme molecule and the reaction intermediate (Siegel et al., 2015). Following the above protocol, an artificial metalloenzyme has been redesigned and the binding capacity of the mutant to the coenzyme was increased by 46 times, and the catalytic activity was increased by 4 times (Heinisch et al., 2015). *Bacillus sp.* YM55-1 aspartase (AspB) has also been designed to expand its substrate scope by computationally reshaping the active pocket to accommodate novel substrates using statistics of near-attack conformations (NACs) and Rosetta Enzyme Design for energy calculation. The NAC is an enzyme reaction mechanism-based conformation that was defined based on the quantum mechanical modeling with distances between reacting atoms smaller than the van der Walls contact distances, angles between reacting atoms deviating less than 20° and all hydrogen bonds anchoring the transition states persistent in the MD simulations (Hur and Bruice, 2003). The reaction mechanism of AspB was first examined to define the near-attack conformations. The Rosetta Enzyme Design was then applied to reshape active pocket to accept alternative groups of novel substrates. Since the novel substrates have the same β-carboxylate as the native substrate, but a different group instead of α-carboxylate that the native substrate possesses, the Rosetta design was constrained to maintain the substrate in the NAC, and to preserve the interaction network of the β-carboxylate whereas the α-carboxylate binding pocket was redesigned to accommodate new substituent groups. This procedure generated enzyme variants that effectively catalyzed asymmetric addition of ammonia to substituted acrylates, affording enantiopure β-aminobutanoic acid, β-aminopentanoic acid, β-asparagine and β-phenylalanine (Li et al., 2018). Recently, the AspB was further designed to achieve addition of a variety of nucleophilic amines to unsaturated acids with numerous different non-canonical amino acids successfully produced at high yield and enantioselectivity (Cui Y. et al., 2021). Combination of MD simulation and semiempirical QM calculations also enables design of enzyme with improved activity towards non-native substrate. Attack conformations of the substrate of amine transaminase from *Chromobacterium violaceum* were first determined by extended MD simulations. Then, the substrate access tunnel was subjected to *in silico* mutagenesis. The random variants were screened and ranked employing the *QzymeDesigner* tool embedded with semiempirical QM calculations based on the minimal interaction energies and substrate distances to the catalytic site. Two double mutants designed by the modelling exhibited >200-fold enhanced activities in the conversion of bulky 1-phenylbutylamin (Voss et al., 2018).

### 6.2 Enantioselectivity

Although fewer successful cases were reported for engineering enzyme enantioselectivity using computational protein design than engineering activity, the tools used for designing enzyme variants with enhanced activity are still useful for engineering enantioselectivity. For example, RosettaDesign has been used to design a limonene epoxidase (LEH) that generates (*R,R*)-products or (*S,S*)-products with high stereoselectivity (Wijma et al., 2015). The study aimed to design mutations that formed a catalytic pocket in which the substrate was selectively positioned in one of the two catalytic orientations. The sequence space was generated by making mutations at eleven sites around the active center to any of the nine hydrophobic amino acid residues. The substrate was placed in the active center of limonene epoxide hydrolase either in a pro-*SS* or pro-*RR* attack position. The sequence space was then searched by RosettaDesign to find substrate-bound structures with strong binding affinity. The obtained variants were then further evaluated by NAC frequency calculated through analysis of MD simulations trajectories. Ten designs with the highest predicted pro-*RR* selectivity together with 27 pro-*SS* designs were then expressed and tested for activity as well as enantioselectivity. It turns out that 28 of 33 active variants showed the enantioselectivity that they were designed for (Wijma et al., 2015). Recently, a similar approach was applied to improve stereoselectivity of CYP105AS1, a cytochrome P450 from *Amycolatopsis orientalis* (Ashworth et al., 2022)*.* Previous directed evolution has generated mutants of CYP105AS1 that can catalyze the hydroxylation of substrate compactin to yield pravastatin, an LDL cholesterol-lowering drug, but with modest selectivity of an *ee* value lower than 90%. The RosettaDesign search space was first selected by making calculations at 14 selected positions guided by diversity of the aligned sequences and active site topology. Based on the 9052 generated low-energy designs binding substrate in a desired *pro*-*S* conformation, six positions were excluded from further optimization as most of the designs at these positions maintained the wild-type residues or an amino acid with similar properties. The other eight positions were then targeted with sampling of all 20 amino acids by Rosetta CoupledMoves and 3417 designs binding substrate in *pro*-*S* conformations were obtained. Yasara docking simulations were then carried out with four low-energy Rosetta designs obtained to confirm the role of the selected mutations in stereo discrimination. Finally, based on Rosetta calculation, docking results and the visual inspection, mutations at positions F76, P80, T95, V180, and T286 were selected for introduction at the template. The best double mutant T95F/V180M gave perfect production of the desired epimer with selectivity >99% (Ashworth et al., 2022). Computational design based on calculation of binding energy was also applied to design enzyme variants with improved diastereoselectivity. It was assumed that for the L-threonine aldolase (LTA) from *Bacillus nealsonii* (*Bn*LTA), the *syn*-configuration was formed by substrate 4-methylsulfonyl benzaldehyde (MTB) attack of the α-carbon atom of pyridoxal phosphate (PLP)−glycine through the *syn* path, and the *anti*-configuration was formed by attack of the α-carbon atom of PLP−glycine through the *anti* path (Zheng et al., 2020; Zheng et al., 2023). Hence, in order to improve diastereoselectivity of *Bn*LTA towards *syn*-configuration product, the variants in *syn*-path with improved binding affinity to substrate and the variants in *anti*-path with reduced binding affinity to substrate were virtually screened by calculating binding energy using tool of Discovery Studio. Combination of variants from two access tunnels led to the best variant with the *de* value of L-syn-3-[4-(methylsulfonyl) phenylserine] reaching 93.1% (87.2%_conv_) (Zheng et al., 2020).

## 7 Conclusion and prospects

The biggest challenge of rational design is to establish accurate models and methods for predicting mutations that significantly improve the properties of enzyme molecules based on a clear understanding of the relationship between enzyme structure and function. Although it is still far away from fully understanding the relationship between protein structure and function, the strategies discussed above can still help to greatly simplify enzyme engineering. The strategy based on multiple sequence alignment is more straightforward and user-friendly compared to other methods as it does not require the protein structure and clear understanding about the catalytic mechanisms. The strategies based on steric hindrance and remodeling interaction network would be more accurate and widely applied for engineering enzymes than the strategy based on solely the sequence since the researchers already had, to a large extent, a clear understanding about the enzyme mechanism before proposing potential mutations. The strategy based on dynamics modification is a cutting-edge area, which is promising but challenging due to the complex relationship between protein dynamics and enzyme function. Computational protein design has not been widely applied in enzyme engineering as current tools were not quite user-friendly and accurate. In the future, the wide applications of rational protein design strategies in enzyme engineering will most likely benefit from the further development of computational protein design tools and application of machine learning.

The application of machine learning in protein engineering includes the construction and optimization of models based on the existing enzyme mutant sequence and functional data, and the subsequent use of the models to predict new mutants with improved effects. Although machine learning has achieved several successful cases in enzyme engineering (Yang et al., 2018; Alley et al., 2019; Yang et al., 2019; Thean et al., 2022), there is currently no universal method for a wide range of applications. Additionally, deep learning method has been applied for designing functional proteins, which is promising to construct enzymes with novel functions (Wang J. et al., 2022). However, when machine learning was applied in protein engineering, data scarcity was the foremost challenge as it takes much time, cost and resources to experimentally collect the activity data of dozens to hundreds of enzyme variants towards different substrates (Mazurenko et al., 2019). In the future, with the help of automatic devices used for high-throughput DNA assembly, protein expression, purification, and enzyme characterization, a large number of experimental data will be collected automatically in a standard form, which is expected to accelerate the wide application of machine learning in protein engineering.
